# Approaches to altering particle distributions in cryo-electron microscopy sample preparation

**DOI:** 10.1107/S2059798318006496

**Published:** 2018-05-18

**Authors:** Ieva Drulyte, Rachel M. Johnson, Emma L. Hesketh, Daniel L. Hurdiss, Charlotte A. Scarff, Sebastian A. Porav, Neil A. Ranson, Stephen P. Muench, Rebecca F. Thompson

**Affiliations:** a School of Molecular and Cellular Biology, Faculty of Biological Sciences and Astbury Centre for Structural and Molecular Biology, University of Leeds, Leeds LS2 9JT, England; b School of Biomedical Sciences, Faculty of Biological Sciences and Astbury Centre for Structural and Molecular Biology, University of Leeds, Leeds LS2 9JT, England; c School of Chemistry, Faculty of Mathematics and Physical Chemistry and Astbury Centre for Structural and Molecular Biology, University of Leeds, Leeds LS2 9JT, England; d National Institute for Research and Development of Isotopic and Molecular Technologies, 67-103 Donat, 400293 Cluj-Napoca, Romania

**Keywords:** cryo-electron microscopy, single-particle sample preparation, single-particle analysis

## Abstract

Cryo-electron microscopy (cryo-EM) can now be used to determine high-resolution structural information on a diverse range of biological specimens. Recent advances have been driven primarily by developments in microscopes and detectors, and through advances in image-processing software. However, for many single-particle cryo-EM projects, major bottlenecks currently remain at the sample-preparation stage; obtaining cryo-EM grids of sufficient quality for high-resolution single-particle analysis can require the careful optimization of many variables. Common hurdles to overcome include problems associated with the sample itself (buffer components, labile complexes), sample distribution (obtaining the correct concentration, affinity for the support film), preferred orientation, and poor reproducibility of the grid-making process within and between batches. This review outlines a number of methodologies used within the electron-microscopy community to address these challenges, providing a range of approaches which may aid in obtaining optimal grids for high-resolution data collection.

## Abstract 2

This paper describes different approaches that cryo-EM users can take to improve the quality of their sample distribution and ice for high-resolution single-particle cryo-EM.

## 1. Introduction   

The last ∼5 years have seen significant developments in the electron-microscopy (EM) field, with a rapid expansion in the use of single-particle approaches to determine high-resolution structures, including those of macromolecular complexes, membrane proteins and ribosomes (Nguyen *et al.*, 2015 ▸; Fitzpatrick *et al.*, 2017 ▸; Plaschka *et al.*, 2017 ▸). From large viruses which may be over 80 nm in diameter and many tens of megadaltons to small soluble proteins of less than 150 kDa, single-particle analysis can offer insights into the structure and function of a diverse range of macromolecular complexes (Khoshouei *et al.*, 2017 ▸; Hesketh *et al.*, 2015 ▸; Vinothkumar *et al.*, 2016 ▸; Rawson *et al.*, 2018 ▸). Through developments in direct electron-detector technology, improved microscope hardware and more advanced image-processing algorithms, the expected resolution from EM has significantly changed, with 831 structures deposited in the EMDB with a resolution of <4 Å since 2013, compared with 18 in the preceding ten years (as of January 2018).

**Figure 1:**
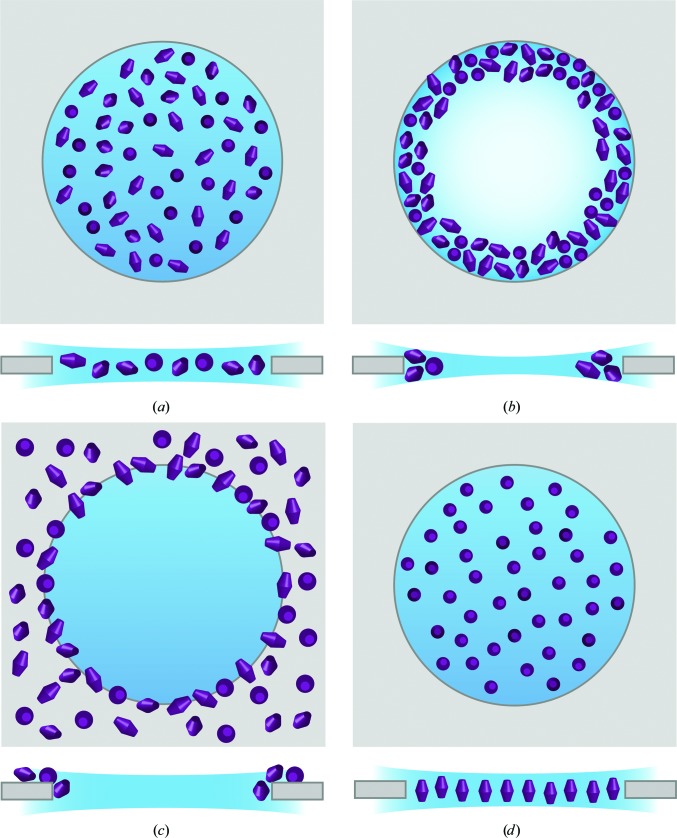
Schematic representations of macromolecular complexes distributed in a vitreous ice layer. Top panels, views from the top; bottom panels, views from the side. (*a*) Ideal vitrified sample exhibiting well dispersed particles adopting random particle orientations. (*b*) Thinning of the ice in the centre of the hole pushes particles towards the carbon edge, excluding any from the middle and causing particle aggregation. (*c*) Specimen exhibits high affinity for the support and is excluded from the holes. (*d*) Particles adopt a preferential orientation.

Despite these advances, substantial barriers to high-resolution data collection remain for many projects. One such barrier is the inefficiency of sample preparation; the precise methodology is often different for each sample studied, somewhat akin to the crystallization step in X-ray crystallo­graphy. The preparation of high-quality grids for cryo-EM analysis relies on being able to preserve the specimen in a vitreous thin film, with the particles adopting an even distribution and ideally exhibiting random orientations within the ice layer (Fig. 1 ▸ *a*). This is achieved by ‘plunge freezing’, a methodology developed in the 1980s, when it was demonstrated that water could be frozen into an amorphous, noncrystalline, ‘glass-like’ state by plunging into liquid ethane (Dubochet *et al.*, 1988 ▸). This methodology is so central to the cryo-EM technique that its development has been recognized by a share in the Nobel Prize in Chemistry in 2017 for Jaques Dubochet.

The standard plunge-freezing workflow for single-particle analysis typically consists of (i) selecting an EM grid and support film, (ii) rendering that support film hydrophilic, (iii) applying the sample to the EM grid and forming a thin film, within which the sample is suspended, by blotting and (iv) vitrifying the sample by plunge freezing into a cryogen such as liquid ethane. While this overall process can be applied to a wide range of biological specimens, there are many variables that can affect either the quality of the ice on the grid, the distribution of particles within that ice, or both. Common problems encountered with plunge-frozen specimens that represent a barrier to high-<!?bk [?tjl=100mm]$262#[?tjl]$w>resolution imaging include protein aggregation, denaturation and the disassociation of labile complexes, particles adopting a preferred orientation, a preference for, or aggregation on, the support film and/or the edges of the holes in that film, and non-uniform distribution across the grid (Figs. 1 ▸ *b*, 1 ▸ *c *and 1 ▸ *d*).

With an ideal specimen, the macromolecules in question are monodisperse and adopt random orientations within the vitreous ice layer, resulting in a monotonic distribution of angular projections. In reality, few specimens present entirely random orientations owing to the interactions that they make with the air–water interface, neighbouring particles and/or the support film. Indeed, in some cases sample interactions at the air–water or support–water interface can result in the specimen adopting specific strongly preferred orientations. This results in a biased distribution of angular projections, and consequently a proportional under-sampling of some Fourier components within the final reconstruction. This is especially problematic for particles with low or no symmetry, and can impact the overall resolution and quality of the map (missing views often present as a ‘smearing’ of the density), and in extreme cases can lead to an entirely incorrect density map. While preferred orientation can be compensated for at the time of data collection by collecting tilted images (Tan *et al.*, 2017 ▸), in our experience microscope stages are less stable at higher tilts, specimen thickness is greater and a defocus gradient is introduced across each micrograph, leading to a deterioration in both the quality and the quantity of the data recorded. Preferred orientation is therefore better tackled at the sample-preparation stage where possible.

Here, we present a selection of methods from our experience and the wider literature that can be used to improve the quality of plunge-frozen cryo-EM samples. Together, these form a ‘toolbox’ of approaches which cryo-EM practitioners can use to optimize the quality of their samples.

## 2. Starting with the sample   

If a macromolecular complex is unstable, intrinsically dis­ordered or has buffer components that are incompatible with plunge freezing, it is futile to attempt to produce optimized cryo-EM grids with a good particle distribution. The best recipe for success in a cryo-EM experiment is proper characterization of the sample before attempting plunge freezing. Biochemical and biophysical tools such as SEC-MALLS, negative-stain EM, thermal melting temperature determined by circular-dichroism/thermal stability assays, and if available, binding/activity assays are all valuable ways of assessing sample suitability and stability for cryo-EM. Negative stain is a powerful tool for assessing sample heterogeneity, and where possible we would always recommend it as an initial characterization step on the pathway to a cryo-EM structure.

A major challenge when studying biological systems using single-particle cryo-EM is that there is usually very little difference between the contrast of the supporting buffer and the protein since both are primarily made up of nuclei with similar atomic numbers (O, H, C and N). Therefore, the greater the concentration of salts and organic molecules such as glycerol, the smaller the contrast difference between the protein specimen of interest and the surrounding buffer, the poorer the signal-to-noise ratio in the images and the harder it is to assign angles accurately during image-processing steps.

Glycerol is commonly added to buffers to stabilize proteins, for example when freezing samples for storage. For high-resolution cryo-EM studies the inclusion of glycerol should be avoided if at all possible, as it greatly reduces contrast. With a specimen containing 50% glycerol, the solvent density is 1.181 g cm^−3^ at 72 K, which is very close to the average density of protein molecules at 1.35 g cm^−3^ (Karuppasamy *et al.*, 2011 ▸). In our experience >5% glycerol can affect the contrast in micrographs to the extent that it significantly degrades the quality of the resulting data, especially for smaller (<500 kDa) proteins. Similarly, the salt concentration required to stabilize the macromolecular conformation varies significantly between different samples. However, as a general rule it is best to keep the concentration of salt as low as possible, ideally below ∼300 m*M*, in order to maximize contrast. The pH may also play an important role and in crystallization experiments this parameter is often screened over a broad range, typically from pH 4.0 to 9.0. However, this diversity in pH is not seen in many EM experiments and is often, mistakenly, not factored into initial sample-screening protocols. Biochemical tools can be used to identify optimal buffer conditions for structural analysis. A number of approaches exist for screening the effect of pH and other buffer components on protein stability, with, for example, ProteoPlex allowing rapid screening of the effect of multiple parameters on complex stability (Chari *et al.*, 2015 ▸). Mass spectrometry is also playing an increasing role in finding optimized solution conditions for structural determination of complexes (Liko *et al.*, 2016 ▸). Having comparable buffers for both biochemical characterization and structure determination makes the interpretation of the data more straightforward and so, where possible, using identical buffers for both is preferable.

### 2.1. Optimizing a sample for cryo-EM   

#### 2.1.1. Surfactants   

The thin film formed during the blotting process is a harsh environment for macromolecular complexes. The film itself is only ∼10–90 nm thick, and so in the seconds between the process of blotting and plunging into cryogen Brownian motion will cause the macromolecules to collide with the air–water interface thousands of times per second (Trurnit, 1960 ▸). It has long been known that the forces that macromolecular complexes encounter at the air–water interface may cause the dissociation of labile complexes and even the denaturation of protein domains (Taylor & Glaeser, 2008 ▸; Glaeser & Han, 2017 ▸; Postel *et al.*, 2003 ▸). There have been some innovative specimen-specific approaches to protect samples from the forces present at the air–water interface, such as the design of a three-dimensional DNA origami scaffold to encompass the transcription cofactor p53 (Martin *et al.*, 2016 ▸), but for most samples this approach is not practically achievable. A more general approach is to add surfactants such as dodecylmaltoside to help to prevent denaturation at the air–water interface, even for soluble proteins (Glaeser *et al.*, 2016 ▸). Such surfactants can protect against dewetting and rupture of the surface, akin to the ability of a ‘soapy’ solution to make bubbles. When a surfactant is added in sufficient quantities (dependent on the surfactant) to the sample of interest during the plunge-freezing process a monolayer of surfactant can form at the air–water interface, effectively shielding the specimen from contact with the air–water interface (Glaeser & Han, 2017 ▸).

#### 2.1.2. Cross-linking   

Many protein complexes display heterogeneity in their stoichiometry through weak or transient binding of partner proteins. During grid preparation, contact with the strong forces at the air–water interface can result in the dissociation of such protein complexes, generating a significant, grid-induced, compositional heterogeneity. To overcome this, cross-linking can be used to stabilize complexes. The GraFix methodology chemically cross-links a sample within a density gradient, allowing the purification of monodisperse chemically stabilized complexes by ultracentrifugation (Stark, 2010 ▸). This approach can significantly reduce the heterogeneity associated with particle dissociation and therefore is a powerful tool for the study of heterogeneous complexes (Du *et al.*, 2014 ▸; Nguyen *et al.*, 2013 ▸). The basic GraFix methodology has been adapted further for membrane proteins with the development of GraDeR, which can remove free detergent from samples of membrane-protein complexes whilst improving the stability of fragile multi-subunit complexes through cross-linking (Hauer *et al.*, 2015 ▸). Both of these techniques use cross-linking and subsequent ultracentrifugation in a density gradient, which is followed by a buffer-exchange step to remove the gradient-forming molecule from the sample prior to EM grid preparation. To avoid ultracentrifugation steps altogether, cross-linking in solution could also be considered (Engel *et al.*, 2016 ▸; Kang *et al.*, 2017 ▸).

#### 2.1.3. Positive controls and labelling   

One of the greatest challenges when working with a new sample, especially one which is relatively small and/or for which the stoichiometry, and so the expected size, is not accurately known, is picking out ‘particles’ from background features/noise. It can be extremely challenging to assess both the ice thickness and the particle concentration. In this situation, we commonly mix the specimen of interest with a well characterized protein, for example F-actin, or *Tobacco mosaic virus* (TMV). This offers two advantages. The first is that when screening the grids one can more easily assess whether the ice thickness is appropriate. In the case of TMV, given its high symmetry it can also be used for downstream assessment of overall grid quality by processing these particles separately and looking at the final resolution of the resulting density map. A second advantage of adding a filamentous protein is that they can aid the creation of more uniform thin films across the grid. If attempting this approach, be aware that in some cases the specimen of interest interacts with the second sample.

As a further means of assessing specimen quality and also for identifying whether a particular subunit is present (and its location), there are a number of different labelling approaches that can be used. Antibody binding has the advantage of adding significant mass to the sample, which is useful for smaller (<100 kDa) specimens (Wu *et al.*, 2012 ▸), as exemplified for an ABC heterodimeric exporter (Kim *et al.*, 2015 ▸). Alternatively, tagging can be used to map the location of inhibitor-binding sites by increasing the bulk of the bound compound such that it can be visible even in lower resolution negative-stain data (Muench *et al.*, 2014 ▸). Another approach is to use gold labels or quantum dots, which are clearly visible even in the raw images (Low *et al.*, 2014 ▸; Gold *et al.*, 2014 ▸). These can be conjugated to antibodies, or 5–20 nm gold particles can be used which bind to polyhistidine tags on proteins and can be useful in quickly assessing protein distribution in the raw images. Care must be taken to remove the background gold particles so as not to overestimate the number of particles per image. This has also been extended to labelling proteins within vesicles to quantify the protein distribution within proteoliposomes. Consideration must also be given to any downstream processing, where the strong signal from gold clusters can dominate the alignment procedure, making image processing more challenging. However, this can be a powerful approach for looking at membrane-protein distribution upon the surface of a proteoliposome. Other labelling approaches include the use of engineered tags such as green fluorescent protein (Roberts *et al.*, 2012 ▸) and the DID–Dyn2 tag (Flemming *et al.*, 2010 ▸).

## 3. Approaches for altering the particle distribution   

### 3.1. Support films   

**Figure 2:**
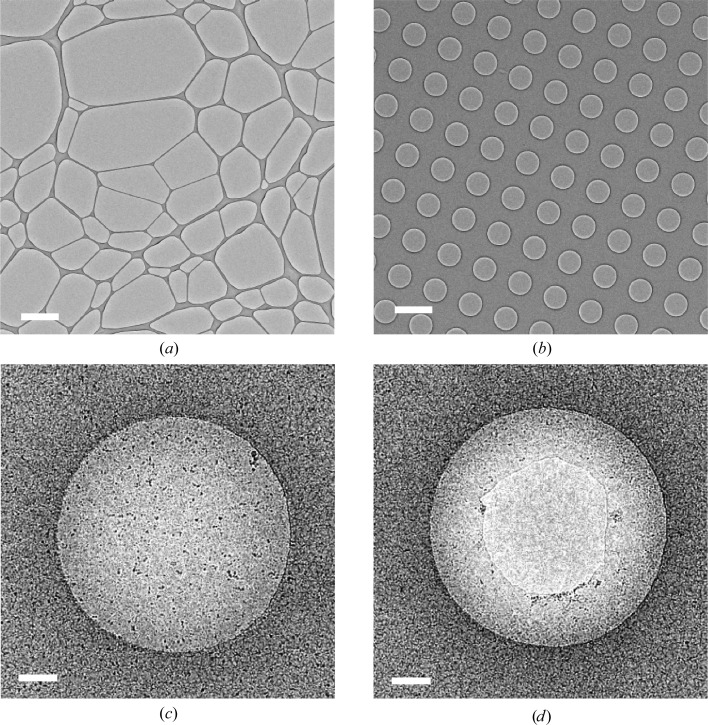

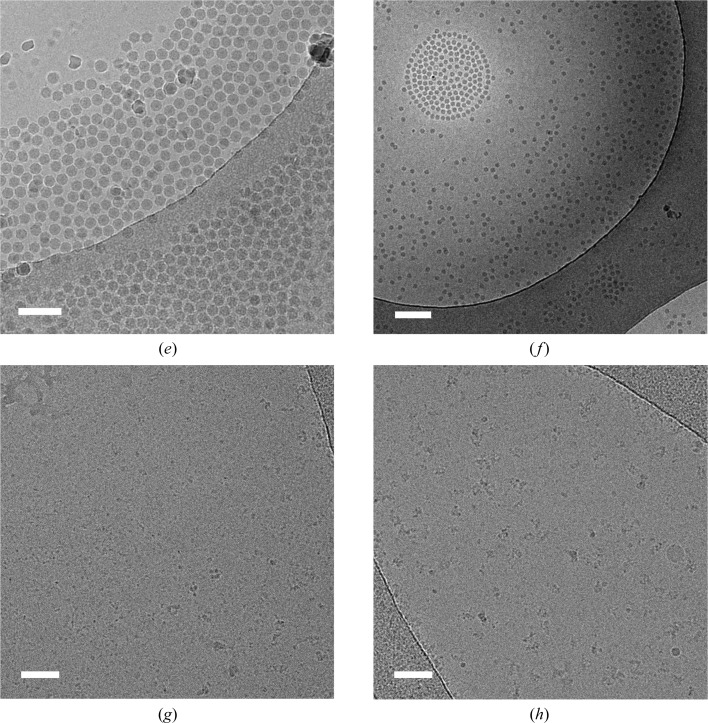
Effect of hole size on thin films and particle distributions. (*a*) A lacey grid with an irregular distribution of hole sizes and shapes. (*b*) A Quantifoil R1.2/1.3 with a regular distribution of evenly sized holes. (*c*) An even distribution of ice across the hole as seen for the R1.2/1.3 grids. (*d*) An example of broken substrate in the hole centre. (*e*) Thin ice in the centre of the hole excluding the virus and causing it to clump towards the edge of the hole. (*f*) Virus particles forming a semi-ordered array in the area of thin ice. (*g*) An example of a 750 kDa multiprotein complex disassociating in the large holes of lacey carbon. (*h*) The same multiprotein complex remains intact and with a range of angular distributions in the smaller holes of lacey carbon. Scale bars: (*a*), (*b*) 2 µm; (*c*), (*d*), (*f*) 200 nm; (*e*) 100 nm; (*g*), (*h*) 50 nm.

Typically, a cryo-EM sample is applied onto a 3 mm metal mesh grid, with a support film of amorphous carbon layered over the top. A range of metals can be used for the metal mesh, including gold, nickel, molybdenum and, most commonly, copper, with 200–400-mesh grids being the most prevalent choice for single-particle projects. The support film is typically perforated with small (1–2 µm) holes, which can be arranged in an irregular or regular array (Figs. 2 ▸ *a* and 2*b*). Both kinds of support film can be either made in-house (Marr *et al.*, 2014 ▸; Lünsdorf & Spiess, 1986 ▸) or purchased commercially, such as Quantifoil and C-flats.

The choice of grid can also have downstream effects on the ease, speed and quality of automated data collection. Conventional amorphous carbon has proven to be an invaluable support film for high-resolution cryo-EM, but it is prone to bending and deformation as a result of exposure to the electron beam (Brilot *et al.*, 2012 ▸). This results in beam-induced specimen motion which, to some extent, can be rectified using motion-correction algorithms (Scheres, 2014 ▸; Rawson *et al.*, 2016 ▸). Minimizing motion at the specimen level is preferable, especially if very high resolution is the target of the study. Recently, an all-gold cryo-EM specimen support has been developed and commercialized as UltrAu­Foil Holey Gold Films. Such gold supports have been shown to significantly reduce substrate motion during illumination with the electron beam (Russo & Passmore, 2014*b* ▸). Other support films such as silicon carbide (Yoshioka *et al.*, 2010 ▸) and amorphous titanium–silicon glass (Rhinow & Kühlbrandt, 2008 ▸) have also been reported to improve the quality of data collection through reducing beam-induced movement.

#### 3.1.1. Hole shape and size   

A plethora of hole sizes and shapes are available for EM grids. The physical properties of the hole, such as the diameter and thickness of the carbon film, have an effect on the formation of the aqueous thin film and the distribution of sample within the ice layer (Cho *et al.*, 2013 ▸). The thin films formed during the blotting process are unstable, and reproducibly controlling their thickness is extremely challenging. Once the thickness of the aqueous film drops below ∼100 nm, van der Waals forces act to further thin the film until complete dewetting occurs (Glaeser *et al.*, 2016 ▸). While the precise physical forces that are present during blotting and plunge freezing are poorly characterized, the practical upshot is that it is impossible to achieve a perfectly even thin film of ice across an entire 3 mm grid; some areas of the grid will become air-dried, whilst at the same time adjacent areas may remain too thick.

In our experience, it tends to be easier to achieve good ice thickness for high-resolution imaging with smaller hole sizes. When using larger holes, we often observe thinner ice in the centre of the hole and thicker ice at the hole edge, which often has the effect of excluding particles from the middle of the hole and crowding particles against the carbon (Figs. 2 ▸ *c*–2 ▸ *f*). In extreme cases, a physical hole in the middle of the thin film leaves a ‘halo’ suspended around the edge of the hole. This effect appears to be more prevalent when detergents are present in the buffer. Such incomplete ice layers are not optimal as the unsupported substrate will exhibit more motion compared with a situation where ice is fully suspended across the hole.

One example of this is an ∼480 kDa membrane protein, cytochrome *bc*_1_, in 25 m*M* potassium phosphate pH 7.5, 100 m*M* NaCl, 0.5 m*M* EDTA, 0.015% DDM buffer (Amporndanai *et al.*, 2018 ▸). Initially, R2/2 grids were prepared and we observed that in the majority of holes the resulting particles were clumped together around the carbon edge, with many holes having a physical break in the centre (Fig. 2 ▸ *d*). During the process of grid optimization, R1.2/1.3 grids were chosen, with the resulting particles displaying a significantly improved distribution in the holes, with a reduced number of holes with a broken centre. The improved particle distribution from these grids with smaller holes enabled the straightforward optimization of autopicking parameters. This resulted in a map of improved resolution that was achieved on a shorter timescale.

The properties of the thin film formed during plunge freezing can have a striking effect on the stability of a macromolecular complex. Manipulating the size and shape of the hole, and therefore the behaviour of the thin film, may be a mechanism to alter the structural dynamics of such complexes. One example of this effect is an asymmetric multi-protein complex of 750 kDa in 40 m*M* Tris–HCl pH 7.4, 150 m*M* NaCl. On Quantifoil R1.2/1.3 and R2/2 grids the complex appeared to have completely disassociated. However, in the small holes of a lacey carbon grid it appeared to be intact, and a broad distribution of views were present (Fig. 2 ▸ *h*). This observation has been repeated across multiple batches of both protein and grid preparation. On the lacey carbon grid, the complex also appears to be dissociated in the larger holes, with no views corresponding to the intact complex observed (Fig. 2 ▸ *g*). We rationalized that the cause of this effect on particle distribution could be owing to the complex becoming dissociated or denatured at the air–water interface. In the case of our particular problem complex, we suggest that the forces acting on the protein complex are weaker in thicker films (*i.e.* those suspended over the smaller holes of lacey carbon) and this preserved the complex intact.

If it is suspected that a complex is falling apart, screening conditions with lacey and Quantifoil Multi grids can offer a quick way to assess a range of hole sizes without having to prepare multiple grid types. However, for automated data acquisition with EPU (FEI) it can be advantageous to collect data using a regular array of holes, as beam deflectors can be used to collect multiple images around a hole, significantly speeding up data acquisition.

#### 3.1.2. Continuous support films   

**Figure 3:**
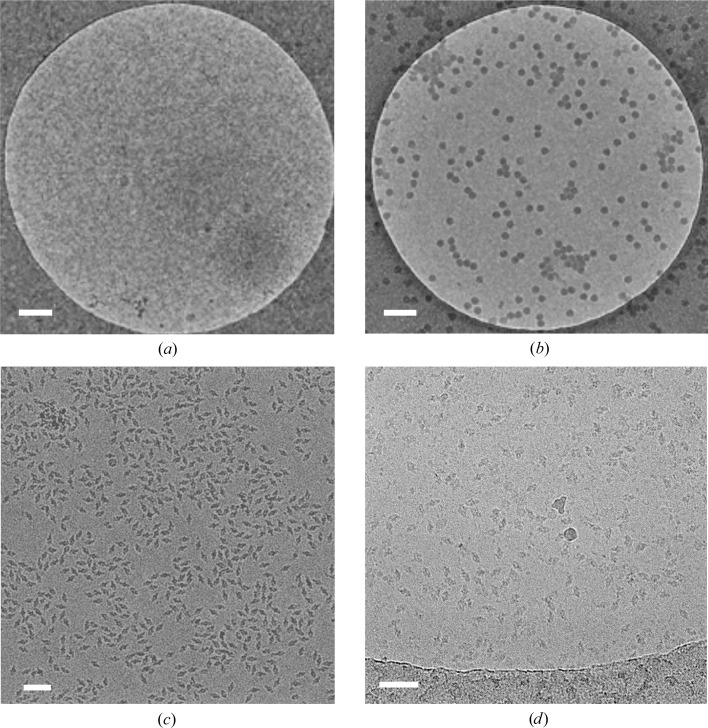
The use of continuous support films. (*a*) Holey grid showing few virus particles in the vitrified ice. (*b*) Continuous carbon grid prepared with the same concentration of virus as in (*a*), showing a drastic increase in the number of virus particles observed. (*c*) Representative micrograph of a holey grid showing the extreme preferred orientation of β-galactosidase particles. (*d*) Representative micrograph of a continuous graphene oxide grid with a significantly improved angular distribution of β-galactosidase particles. Scale bars: (*a*), (*b*) 200 nm; (*c*), (*d*) 50 nm.

One phenomenon that is commonly experienced by cryo-EM researchers is to observe that their specimen has a strong affinity for the carbon film, resulting in a poor distribution of particles in the thin film suspended between the holes. For some specimens, an easy and extremely effective solution is to use grids with a continuous thin (<5 nm) carbon film (Figs. 3 ▸ *a* and 3 ▸ *b*). These can both be made in-house by floating a thin film of carbon onto grids or directly sputtering carbon onto a collodion surface, or purchased commercially, including lacey supports with a 3 nm continuous carbon layer from Agar Scientific and Quantifoil. This approach was used to improve the particle distribution of V-ATPase and allow more efficient data collection by EPU on Quantifoil grids rather than the lacey grids that were originally used (Rawson *et al.*, 2015 ▸).

Imaging through a continuous carbon layer has a detrimental effect on the signal-to-noise ratio and makes particles more difficult to align during image-processing steps; therefore, thin carbon films work best on large, >500 kDa macromolecular complexes, including icosahedral viruses. Moreover, the continuous carbon layer can make it difficult to distinguish whether there is vitreous ice present when assessing the grids in low-magnification mode, although this tends to become readily apparent when taking high-magnification (1–2 Å per pixel) images as areas with no ice become visually radiation-damaged much more quickly than areas containing vitreous ice.

For smaller specimens that have an affinity for the carbon support, other more electron-transparent options are available. Graphene is an obvious choice owing to its strength and relative electron transparency; however, unmodified graphene is extremely hydrophobic. Graphene can be made hydrophilic using low-energy hydrogen plasmas (Russo & Passmore, 2014*a* ▸) or chemical modification (Pantelic *et al.*, 2014 ▸). Such modified graphene supports can have a dramatic effect on the particle distribution. Russo and Passmore demonstrated a relationship between low-energy hydrogen plasma dose and the particle number observed on graphene-coated EM grids. In this study, no plasma treatment resulted in very few observed 70S ribosome particles (∼60 particles µm^−2^), while 20 s of treatment improved the particle number approximately tenfold and 40 s of treatment led to a densely packed surface with ∼1900 particles µm^−2^ observed. This method was also successfully applied to tune particle distribution for a number of other specimens, including 80S ribosome, 20S proteasome and apoferritin (Russo & Passmore, 2014*a* ▸). While hydrogenated graphene supports are a powerful tool to tune sample distribution, the specialist equipment required may mean that they are out of reach for smaller EM groups at present.

Graphene oxide is a naturally hydrophilic derivative of graphene and may offer a more convenient alternative to graphene. It is relatively easy to produce graphene oxide-coated grids using standard EM laboratory equipment, and in some cases this can also have a dramatic effect on particle orientation, distribution and on-grid concentration (Pantelic *et al.*, 2010 ▸; Figs. 3 ▸ *c* and 3 ▸ *d*). Flakes of graphene oxide are deposited onto pre-glow-discharged grids, making grid preparation quick and easy. However, the drawback to using flakes of graphene oxide is that even when the process works well, overlapping or wrinkling of flakes limits the usable areas.

EM grids with continuous films can show a partitioning of particles at the air–water and support–water interfaces, with almost none in the ice in the middle (Bharat & Scheres, 2016 ▸). As discussed above, specimens are exposed to physical forces at the air–water interface that can cause protein denaturation. By partitioning most of the particles to the support–water interface, at least partial protection of the specimen from these potentially denaturing forces may be achieved. It is worth noting, however, that there may also be forces at the support–water interface that can affect macromolecule structure, and these are even less well characterized than those at the air–water interface.

#### 3.1.3. Other uses of continuous films   

**Figure 4:**
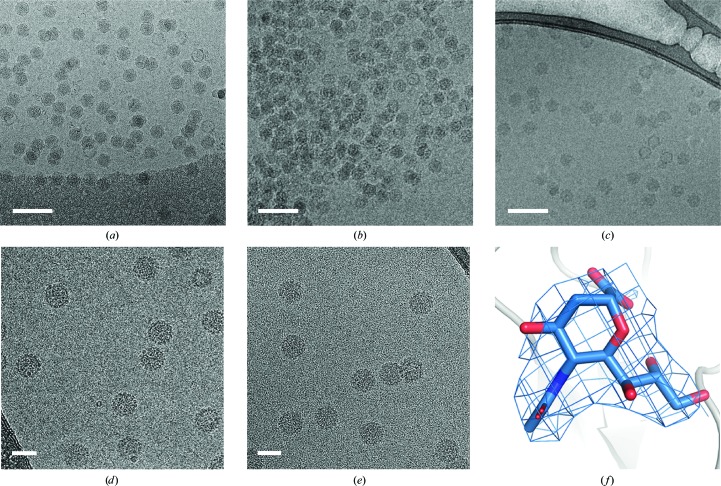
Practical applications of continuous carbon supports. (*a*)–(*c*) Avoiding aggregation by immobilizing the virus prior to the addition of a binding protein, (*d*)–(*f*) grid soaking with low-affinity receptor molecules. (*a*) An example of a virus-only sample distributed evenly across a holey grid. (*b*) Aggregates are observed on a grid after virus and non-antibody binding protein are mixed in solution. (*c*) Virus and non-antibody binding protein complexes are distributed evenly across a grid. Virus sample was applied to a lacey grid with a 3 nm continuous carbon support (Agar Scientific) and the excess solution was blotted away; the binding protein was then applied and the excess was washed away with buffer before blotting and plunge freezing. (*d*) Virus-only sample distributed evenly across a lacey grid with a 3 nm continuous carbon support (Agar Scientific). Owing to the low concentration of the sample and its intractability to concentration, multiple aliquots of virus sample were applied to the grid prior to blotting and plunge freezing. (*e*) Virus and 20 m*M* solution of receptor fragment. Excess virus sample was applied to a grid and blotted away before the concentrated receptor solution was applied. This was left to dwell for 30 s prior to blotting and plunge freezing. (*f*) EM density (2.8σ) and fitted model for a terminal sialic acid present in the low-affinity receptor fragment. Scale bars: (*a*)–(*c*) 100 nm; (*d*), (*e*) 50 nm.

An advantage to using continuous support films is that they allow wash steps to be introduced, similar to those used in negative-staining protocols, or for the sample to be immobilized onto a continuous support before further treatment. A reported phenomenon in cryo-EM sample preparation is that the addition of a receptor or small-molecule inhibitor in solution can cause particles to aggregate (O’Donnell *et al.*, 2009 ▸; Xie *et al.*, 2017 ▸). We have observed this across multiple virus samples with a range of ligands/receptors (Figs. 4 ▸ *a* and 4 ▸ *b*). To mitigate this effect, we utilized a method where virus samples are immobilized onto cryo-grids overlaid with a continuous ultrathin (∼2 nm) carbon film, the excess solvent is blotted away and the desired ligand is then applied prior to blotting and plunge freezing (Fig. 4 ▸ *c*). This methodology has produced several sub-4 Å resolution structures with ligand sizes ranging from 1.5 to 23 kDa and ligand types including carbohydrates and protein receptors (Baggen *et al.*, 2018 ▸). In such cases, both the virus and ligand samples were of very high purity. This method can also be used for small-molecular-mass ligands, in a fashion analogous to crystallographic soaking experiments, where millimolar concentrations of ligands are required to achieve full occupancy of binding sites owing to low-affinity interactions (Figs. 4 ▸ *d*, 4 ▸ *e* and 4 ▸ *f*) (Hurdiss *et al.*, 2018 ▸). The large size of viruses makes them particularly suited to this technique as they are less susceptible to the contrast reduction which results from both the solid support and the presence of excess ligand. However, we envisage that this could also be applied to smaller complexes using a graphene solid support and small-molecule ligands.

Furthermore, labile complexes can be directly cross-linked to a continuous support, preventing the complex from dissociating during thin-film formation. This has been used on the fragile phycobilisome complex (Arteni *et al.*, 2009 ▸). Briefly, this involves the addition of the sample to a carbon-backed EM grid which has been glow-discharged and then adding fresh buffer without any protein followed by a mild cross-linking buffer (0.05% glutaraldehyde) to fix the protein. The grid is subsequently washed through the addition of gradually lower levels of ammonium acetate to remove the glutaraldehyde before addition of the final buffer. One advantage of this technique is that by using carbon-backed grids, the protein distribution is similar to that for negative staining and so initial optimization in terms of protein concentration can be achieved through negative-stain analysis.

Continuous support films and self-assembled monolayers can also be functionalized (Meyerson *et al.*, 2014 ▸). For example, the use of PEGylated gold grids had a striking effect on both the distribution and the angular spread of mammalian respiratory complex I (Blaza *et al.*, 2018 ▸). Continuous support films can be modified with a substance that shows affinity for a target specimen, allowing immobilization, purification and/or concentration of the specimen ‘on grid’. This is potentially an excellent approach for studying complexes of low abundance and purity (Yu *et al.*, 2016 ▸). The use of many different types of affinity layers has been reported, including antibody layers (Yu *et al.*, 2014 ▸), functionalized lipid layers (Benjamin *et al.*, 2016 ▸) and functionalized carbon layers (Llaguno *et al.*, 2014 ▸). Most of these approaches have only resulted in low-to-medium-resolution structures, but recently a virus structure was solved to 2.6 Å resolution by the use of an antibody-based affinity grid (Yu *et al.*, 2016 ▸), demonstrating the potential of such approaches.

#### 3.1.4. Treatment of support films   

Out of the box, carbon support films are hydrophobic, which prevents an aqueous sample from spreading evenly across the grid. Amorphous carbon supports are therefore usually treated with a low-energy plasma within a glow-discharge unit or plasma cleaner. Either in air (glow discharge) or in a defined gas mixture (plasma cleaning), a charge is passed through the residual gas in the chamber, creating ions and radicals which react with the surface, reducing the hydrophobicity and removing surface contamination. Glow-discharge units are relatively cheap and so their use is common, although the treatment that they deliver is not as controllable or reproducible as that of plasma cleaners. Glow discharge in air results in a net negative charge being applied to the carbon film, but chemicals such as amylamine can be introduced into the chamber to produce a net positive charge on the film. Amylamine-treated EM grids have been used in conjunction with a variety of samples including myosin (Milligan, 1987 ▸), the type IV pilus (Craig *et al.*, 2006 ▸), liposomes to prevent extensive binding to the support (Craig *et al.*, 2006 ▸) and the 20S proteasome to overcome the problem of preferential orientation (da Fonseca & Morris, 2015 ▸). Altering both the time and strength of plasma treatment and adding chemicals during the process have been shown to alter particle distribution across a grid (Hidalgo *et al.*, 2001 ▸; da Fonseca & Morris, 2015 ▸).

**Figure 5:**
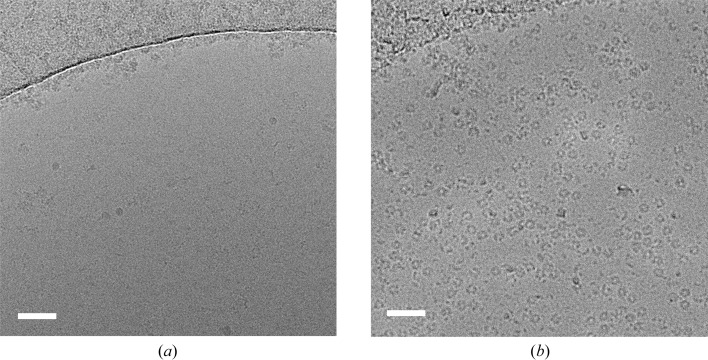
Altering both the time and the strength of plasma treatment can dramatically alter the particle distribution. Representative micrographs of (*a*) very few 600 kDa oligomeric protein complex particles observed on a grid glow discharged using a PELCO easiGlow at 20 mA for 60 s and (*b*) a nice distribution of the same protein complex observed on a grid glow discharged using a Cressington 208 at 10 mA for 90 s. Scale bars are 50 nm.

One example where glow discharge has been shown to impact particle distribution is an oligomeric 600 kDa protein with 12 subunits. In this test, all grids were made in a single freezing session within minutes of each other using the same aliquots of protein and the same batch of Quantifoil grids, while the buffer and glow-discharge parameters were varied. For each of the four different buffer conditions, two grids were made: one glow-discharged using a Cressington 208 glow discharger at 10 mA for 90 s and the other using a PELCO easiGlow at 20 mA for 60 s. For each buffer composition, grids prepared using the Cressington method had well distributed, visible particles, while those prepared using the PELCO easiGlow resulted in very few visible particles (Fig. 5 ▸).

Glow-discharge parameters tend to be some of the last general parameters that we seek to change during the grid-optimization stages. For most projects, as long as the support film is rendered sufficiently hydrophilic for the droplet of specimen to be evenly spread, glow discharge or plasma cleaning does not need to be altered, nor will doing so offer any benefits. However, in specific cases different glow-discharge/plasma cleaning parameters may have a significant effect on sample distribution.

## 4. Blotting apparatus   

Several different types of apparatus are available to aid plunge freezing, including the commercially available FEI Vitrobot, Leica EM GP (and GP2) and Gatan Cryoplunge 3, along with several home-built systems. All commercial systems allow the nominal control of humidity and temperature in the sample chamber to reduce unwanted evaporation from the blotted thin film, as well as to make the process more reproducible. After blotting, given the small volume that remains on the grid, even a small amount of evaporation will result in an unwanted concentration of the specimen and buffer components, which can lead to drastic changes in temperature, ionic strength and pH, and therefore macromolecular complex stability. This can also result in protein aggregation and increased partitioning of the molecules to the air–water interface (Passmore & Russo, 2016 ▸). Besides humidity and temperature control, commercially available systems offer a range of other controllable parameters, such as cryogen temperature control and blotting ‘force’, and have significant differences in their setup. For anyone looking to invest in or upgrade their current system, we have summarized the main characteristics of the blotting instruments that are currently available in Table 1 ▸.

## 5. Non-blotting approaches and future developments   

The blotting of grids using filter paper is a broadly applicable and straightforward approach to making grids that has been suitable for high-resolution structure determination by single-particle cryo-EM. However, there are a number of limitations including, as discussed above, the instability and irreproducibility of thin films and potential evaporation from the film leading to a concentration of buffer/sample components. In addition, the vast majority of the 3–4 µl of specimen applied to the grid is removed by blotting, leaving nanolitre volumes on the grid. Finally, the filter paper contains a number of divalent metals and contaminants. The blotting procedure can typically take 1–6 s, and during this time contaminants from the filter paper can reach levels within the blotted sample which are high enough to be problematic for metal-sensitive proteins such as myosin and when using polymers that are unstable in the presence of high levels of divalent ions (Parmar *et al.*, 2017 ▸; Walker *et al.*, 1994 ▸).

To overcome the problems of using blotting paper, new devices are being developed which remove the need for blotting with filter paper. Spotiton is one such technology that seeks to do this, using ‘self-blotting’ grids combined with a piezo-electric inkjet dispensing system linked to a vitrification device (Jain *et al.*, 2012 ▸; Razinkov *et al.*, 2016 ▸). The thin film is obtained using nanowires, which allow the droplet to spread evenly, resulting in a number of grid squares containing uniform ice (Wei *et al.*, 2018 ▸). As the thin film is more reproducible, less time is spent on screening the grid and finding the areas with optimal ice for data collection. The main advantage of this technique is that it requires only 20–50 nl of sample per grid, meaning that the 3 µl of sample typically applied to one grid using the blotting approach could instead make ∼100 grids (Dandey *et al.*, 2018 ▸). This technique has been used on a range of samples and has resulted in multiple 3–4 Å resolution maps (Zhang *et al.*, 2018 ▸; Scapin *et al.*, 2017 ▸).

An alternative non-blotting technique that has been developed is the cryoWriter, which applies ∼2–20 nl of sample onto the grid by microcapillary action. By directly ‘writing’ the sample onto the grid and controlling the rate of water evaporation, the thickness of the ice layer can be controlled to produce a thin layer of vitreous ice. This method has also been tested using a wide range of samples from membrane proteins to TMV and myosin (Arnold *et al.*, 2017 ▸). As with Spotiton, a significant advantage is the reduced amount of sample that is required to make a single cryo-grid. Such developments are clearly very exciting, but as neither cryoWriter nor Spotiton are currently commercially available access to these emerging technologies remains limited for most cryo-EM researchers.

Developments in time-resolved EM methodology have led to another non-blotting approach. A microfluidic device can be used to generate microdroplets of the sample, which are subsequently sprayed directly onto the grid before freezing with no blotting required. The thickness of the ice can be controlled by changing the distance between the sprayer and grid and altering the spray pressure. Therefore, this approach could be used on different samples which favour a certain ice thickness, thereby improving the reliability of grid-making. Although this method was primarily designed to be used for time-resolved imaging of different samples, it could also be used to also make ‘conventional’ grids, as shown by the 3.0 Å resolution structure of apoferritin (Feng *et al.*, 2017 ▸).

Despite the development of these non-blotting devices, several challenges remain, including overcoming the fundamental problem of degradation of the sample at the air–water interface. However, it seems likely that the greatly increased focus on cryo-EM in recent years will lead to a paradigm shift in sample preparation, as has occurred for both microscope hardware and image-processing software.

## 6. Conclusion   

Where there have been significant changes in the field of EM in microscope hardware, automation of data collection and methods of data processing in the last five years, for the vast majority of cryo-EM users plunge freezing has remained the only method of preparing macromolecular complexes for single-particle analysis. As detailed above, there are a large number of variables which can, in some cases dramatically, alter particle distribution and thin-film formation across a grid during plunge freezing. These, combined with issues of reproducibility of conditions both within and between grid-making sessions, mean that for some samples it can be a significant challenge to find the right conditions and obtain suitable grids for data collection. We see many cases where small changes to a single variable make the difference between unusable and high-quality cryo-EM grids; the ‘dark art’ of grid preparation. For now, researchers’ best hope of optimizing their sample for cryo-EM remains to pay careful consideration to the biochemistry and to robustly characterize the macromolecular complex, followed by, ideally, systematic optimization of cryo-EM grids by trial and error. As methods and technology develop, cryo-EM sample preparation will hopefully become more robust and reproducible.

## Figures and Tables

**Table 1:** Comparison of plunge-freezing instruments

	EM GP	Vitrobot Mark IV	Cryoplunge 3
Manufacturer	Leica	FEI	Gatan
Operating temperature (°C)	+4 to +60	+4 to +60	+4 to +26 (ambient)
Humidity control	Up to 99%	Up to 100%	Up to 100%
Liquefying head	Yes	No	No
Cryogen-pot capacity (ml)	2.5	6.0	4.0
Cryogen temperature control	Yes	No	Yes
Specimen-loading port	Right	Left and right	Front
Blotting	One-sided	Two-sided	One- and two-sided
Blot-force control	Yes	Yes	Yes
Adjustable time delay before/after blotting	Yes	Yes	No
Multiple blotting	Yes	Yes	Yes
Programmable	Yes (up to ten programs)	No	No
Pneumatics supply	N/A	N/A	Nitrogen-gas connection at the back required
Bake-out time	Approximately 2 h†	None‡	None‡
Extra features	Stereomicroscope with LED, adapter for cryo-grid transfer available, blot sensor	Mouse and foot pedal controls available	GentleBlot technology, liquid-nitrogen overflow port, filter-paper punch available

† 1 h bake-out followed by approximately 1 h cooling.

‡ For back-to-back sessions more than one set of cryogen workstations are necessary.
